# Interaction between trouble sleeping and depression on hypertension in the NHANES 2005–2018

**DOI:** 10.1186/s12889-022-12942-2

**Published:** 2022-03-11

**Authors:** Yingjie Cai, Manshuang Chen, Weixia Zhai, Chunhui Wang

**Affiliations:** 1 Department of General Medicine, Shandong Province, Yantai Qishan Hospital, No.62, Huanshan Road, Qishan Street, Zhifu District, Yantai, 264000 People’s Republic of China; 2 Guangzhou University of Chinese Medicine, Guangzhou, 510405 Guangdong Province P.R. China; 3 Department of Intensive Care Unit, Yantai Qishan Hospital, Yantai, 264000 Shandong Province P.R. China

**Keywords:** Hypertension, Trouble sleeping, Depression, Interaction

## Abstract

**Background:**

Hypertension, trouble sleeping and depression, as three major public health problems, were closely related. This study evaluated the independent association of trouble sleeping and depression with hypertension and interaction effect between trouble sleeping and depression on hypertension in Americans.

**Method:**

The data of this cross-sectional study was from the 2005–2018 National Health and Nutritional Examination Survey (NHANES) with hypertension, depression, trouble sleeping and confounding factor information. Multivariate logistic regression model and subgroup analyses of depression severity were conducted to assess the relationship between trouble sleeping and depression on hypertension. Relative excess risk due to interaction (RERI), attributable proportion of interaction (AP) and synergy index (S) were utilized to assess the additive interaction.

**Results:**

A total of 30,434 participants (weighted *n* = 185,309,883) were examined with 16,304 (49.37%) known hypertensive subjects. Compared with participants without trouble sleeping, those with trouble sleeping had a higher risk of hypertension [OR = 1.359 (95% CI: 1.229–1.503)]. We also found the significant association of depression with an increased risk of hypertension [OR = 1.276 (95% CI: 1.114–1.462)], compared with those without depression. Moreover, there was a significant interaction between trouble sleeping and depression on hypertension risk [RERI = 0.528 (95% CI: 0.182–0.873), AP = 0.302 (95% CI: 0.140–0.465), S = 3.413 (95% CI: 1.301–8.951)].

**Conclusion:**

There was a synergistic interaction between trouble sleeping and depression on hypertension, especially the significant synergistic effect between moderate depression and trouble sleeping on hypertension. The results suggested that improving the psychological status and trouble sleeping of patients may be beneficial to the prevention and treatment of hypertension.

## Background

Hypertension, as one of the most common risk factors for cardiovascular disease, is currently defined as systolic blood pressure ≥ 130 mmHg and/or diastolic blood pressure ≥ 80 mmHg, whether for untreated patients or patients taking antihypertensive drugs [1–3]. It affects more than 1.2 billion people worldwide and has become the most critical and costly public health problem [4]. Hypertension remains the most potent predictor of mortality, as a global risk factor for death, disability-adjusted life years and life loss years [5]. Some meta-analyses have shown that hypertension was significantly associated with increased risks of Parkinson's disease, stroke, and other diseases [6, 7]. Consequently, it is necessary and important to study the risk factors and effective predictors of hypertension to reduce the burden on public health.

Sleeping health is increasingly considered a public health problem. Many people suffer from trouble sleeping such as sleep deprivation, poor sleep quality, and sleep disorders, which seriously affect their health [8]. Over the years, several studies suggested that trouble sleeping was associated with high rates of hypertension [9–13]. Studies consistently found that sleeping time was a U-shaped associated with elevated blood pressure [14, 15].

Depression is a mood disorder that leads to a variety of functional physical disorders and loss of interest in daily activities, thereby reducing the quality of life [16]. Studies have shown that individuals with depression had a higher incidence of hypertension [17, 18]. In the United States, data from the National Health and Nutrition Examination Survey (NHANES) reported that patients with self-reported hypertension still had a significant 15% reduction in the relative risk of death compared with the group with self-reported hypertension and depression after adjusting for clinically relevant confounding factors [19].

Therefore, when hypertension and depression or trouble sleeping appear at the same time, two risk combinations may be formed respectively, which highlights the importance of identifying depression or trouble sleeping in subjects with hypertension. Besides, trouble sleeping was associated with an increased psychological risk, such as depression [20]. There were few studies on the direct relationship between the interaction of these two conditions and hypertension. Thus, the purpose of this study was to obtain relevant data from the NHANES, and to study the interaction between trouble sleeping and depression on hypertension.

## Methods

### Study population

We analyzed the data from the NHANES, a representative cross-sectional survey of all non-institutionalized civilian populations in the United States. The NHANES is a major project of the National Center for Health Statistics (NCHS), a part of the Centers for Disease Control and Prevention (CDC) and is responsible for compiling life and health statistics. The survey uses a complex multistage probability sampling design to sample participants, and oversample minorities and older adults so that the results are applicable to other populations [21]. Trained interviewers and examinations use a Computer-Assisted Personal Interview (CAPI) system to collect participants' (self-reported) data. The NHANES data are released in 2-year cycles. To obtain large samples for analysis, we combined seven cycles of the continuous NHANES data from 2005 to 2018. The National Center for Health Statistics Ethics Review Committee granted ethics approval. All individuals provided written informed consent before participating in the study. More information about the NHANES could be obtained at: http://www.cdc.gov/nhanes.

**Fig. 1:**
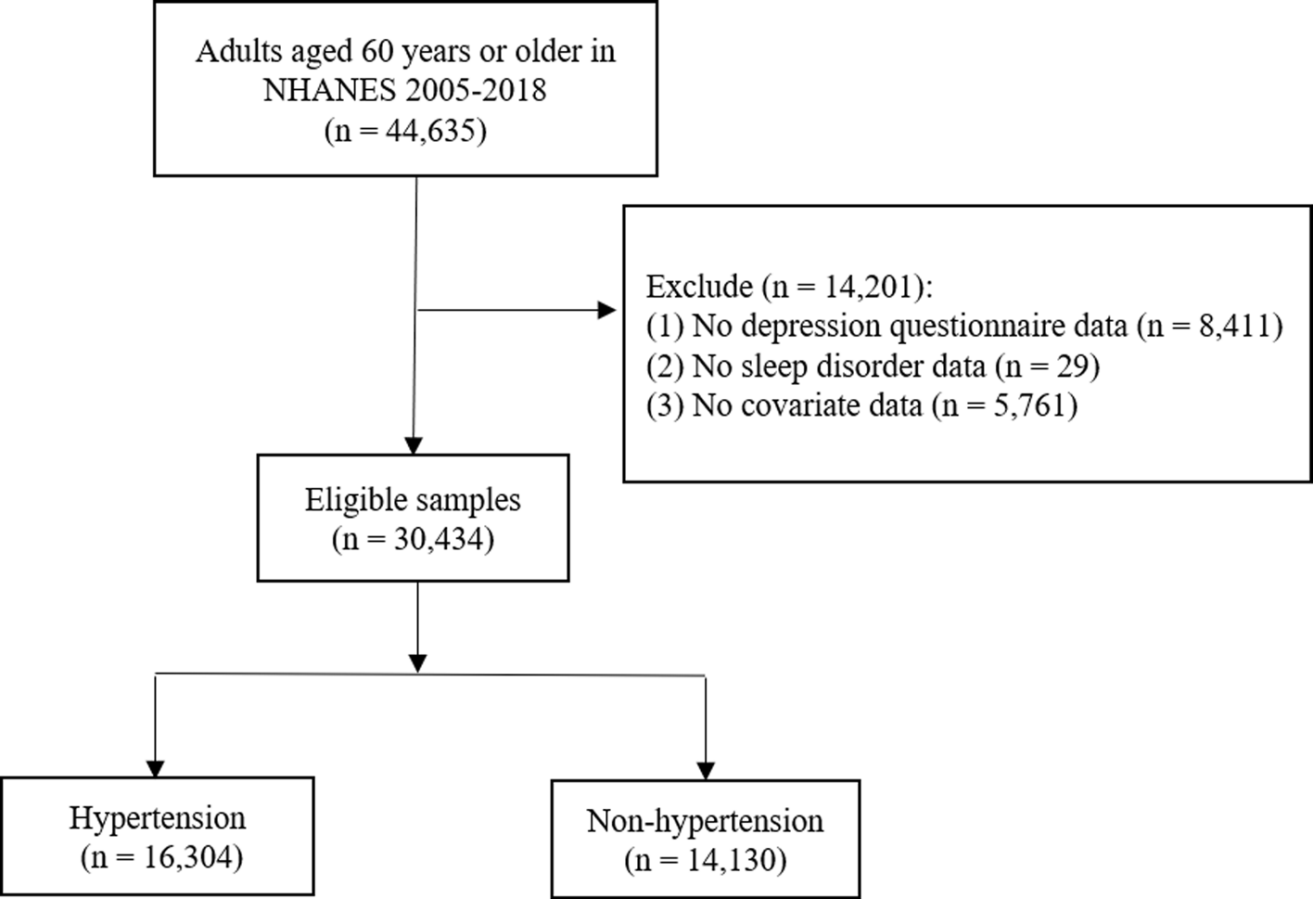
Flowchart of the systematic selection process

Of 44,635 participants extracted from the NHANES database, we excluded those with missing information on depression questionnaire (*n* = 8411), trouble sleeping (*n* = 29), and other covariates (*n* = 5761). Finally, a total of 30,434 participants were included in this study. The flow chart of the systematic selection process is shown in Fig. 1.

### Outcome variable

The outcome variable was that the participant had hypertension. Hypertension was self-reported and blood pressure was measured by the NHANES, which was consistent with methods used in published literature [22–24]. Self-reported hypertension was assessed by an affirmative answer to the question “Have you ever been told by a doctor or other health professional that you had hypertension, also called high blood pressure?” Although this problem was not equivalent to the diagnosis of hypertension, it has been confirmed as screening for hypertension in epidemiological studies [25, 26]. Hypertension measured by the NHANES was the mean blood pressure value of three measurements ≥ 130 mm Hg for systolic blood pressure or ≥ 80 mm Hg for diastolic blood pressure [1].

### Explanatory variables

Trouble sleeping was measured according to the following question “Have you ever told a doctor or other health professional that you have trouble sleeping?” (yes/no). If the participant answered the question positively, then she or he was considered to have trouble sleeping.

Depressive symptoms were tested by the Patient Health Questionnaire (PHQ-9), a 9-item screening instrument that asked the frequency of depressive symptoms in the past two weeks [27]. The total score of PHQ-9 was 0–27 points, of which 0–9 was no depression, 10–14 was moderate depression, 15–19 was moderately severe depression, and 20–27 was severe depression [27]. We used 10 as the cutoff for clinically relevant depression according to previous research [28].

### Covariates

Gender, age and race were self-reported demographic information. The body mass index (BMI) was calculated by dividing the weight of the participant by the square of the height (kg/m^2^). Participants were divided into four categories for marital status (married and or partner/widowed/divorced or separated/never married). Education level was assessed by the question “What is the highest grade or level of school you have completed or the highest degree you have received?” [less than high school/high school graduate or General Educational Development (GED)/some college or above]. Smoking history and alcohol drinking were assessed by the questions “Have you smoked at least 100 cigarettes in your entire life?” and “In any one year, have you had at least 12 drinks of any type of alcoholic beverage?” (yes/no). Doctors diagnosed diseases, including diabetes and stroke, by asking participants “Have you ever been told by a doctor or health professional that you have __?”.

### Statistical analysis

The measurement data were described as mean and standard error (S.E.), and the weighted t-test was used for comparison between groups with hypertension and without hypertension. Categorical variables were analyzed descriptively according to the number of cases and the weighted prevalence [n (weighted%)], and the differences between groups with hypertension and without hypertension were measured by chi-square test. Multivariate logistic regressions analyses were conducted to examine the relationship between trouble sleeping, depression and the interaction items of them and hypertension. The crude Model 1 did not adjust any confounders. Gender and age were adjusted in Model 2. In addition to variables adjusted by Model 2, BMI, race, marital status, education, annual family income, alcohol drinking, smoking history, diabetes, stroke and sleep duration were adjusted in Model 3. Moreover, the additive model was constructed to study whether the interaction was present or not. The additive interaction between trouble sleeping and depression in association with hypertension was measured by whether the estimated joint effect of two factors was greater than the sum of the independent effect of trouble sleeping and depression. Relative excess risk due to interaction (RERI), attributable proportion of interaction (AP) and synergy index (S) were used to assess the additive interaction. When the confidence interval of RERI and AP contained 0 and the confidence interval of S contained 1, there was no additive interaction. Subsequently, the participants were divided into subgroups according to depression severity. All statistical tests were two-sided and completed by SAS v. 9.4 (SAS Institute, Cary, North Carolina) and R v. 4.20 (R Foundation for Statistical Computing, Vienna, Austria) statistical analysis software. *P* < 0.05 was considered as statistically significant.

## Results

### Description of the study population

A total of 30,434 samples (weighted *n* = 185,309,883) were finally included in the study, with the mean age of 47 years, and the mean BMI of 29.06 kg/m^2^. The gender distribution was relatively equal. Of the included participants, 69.06% (*n* = 13,518) were non-Hispanic white, followed by non-Hispanic black (*n* = 6,490, 10.81%), Mexican Americans (*n* = 4,687, 8.15%), other races (*n* = 2,992, 6.86%), and Hispanics (*n* = 2,747, 5.12%). Participants with trouble sleeping accounted for more than a quarter of the study population (27.74%).; 92.46% of the participants had no depression, and the rest had depression, among which moderate, moderately severe and severe depression was 4.76%, 2.00%, and 0.78%, respectively. What’s more, participants with hypertension were 16,304 (49.37%) and without hypertension were 14.130 (50.63%). The baseline characteristics of included individuals are listed in Table 1.

**Table 1:** Group differences between hypertension and non-hypertension groups

Variables	Total [*n* = 30,434 (weighted%)]	Groups		Statistics	*P*
		Non-hypertension (*n* = 14,130)	Hypertension (*n* = 16,304)		
Age, years, Mean (S.E.)	47.30 (0.24)	40.15 (0.25)	54.64 (0.27)	t = -52.95	< 0.001
Gender, n (%)				χ^2^ = 84.39	< 0.001
Male	15,022 (48.98)	6,430 (45.65)	8,592 (52.40)		
Female	15,412 (51.02)	7,700 (54.35)	7,712 (47.60)		
BMI, kg/m^2^, Mean (S.E.)	29.06 (0.09)	27.41 (0.10)	30.77 (0.10)	t = -30.80	< 0.001
Race, n (%)				χ^2^ = 213.48	< 0.001
Mexican American	4,687 (8.15)	2,558 (9.91)	2,129 (6.34)		
Hispanic	2,747 (5.12)	1,425 (6.06)	1,322 (4.15)		
Non-Hispanic white	13,518 (69.06)	6,135 (67.49)	7,383 (70.67)		
Non-Hispanic black	6,490 (10.81)	2,407 (9.09)	4,083 (12.57)		
Other	2,992 (6.86)	1,605 (7.45)	1,387 (6.26)		
Marital status, n (%)				χ^2^ = 969.21	< 0.001
Married and or partner	15,817 (55.81)	7,109 (53.74)	8,708 (57.94)		
Widowed	2,363 (5.60)	429 (2.23)	1,934 (9.06)		
Divorced or separated	4,399 (12.90)	1,657 (10.87)	2,742 (14.99)		
Never married	7,855 (25.68)	4,935 (33.16)	2,920 (18.01)		
Education, n (%)				χ^2^ = 55.34	< 0.001
Less than high school	7,097 (15.48)	3,087 (14.49)	4,010 (16.49)		
High school graduate or GED	7,085 (22.91)	3,024 (21.12)	4,061 (24.75)		
Some college or above	1,6252 (61.61)	8,019 (64.39)	8,233 (58.76)		
Annual family income, n (%)				χ^2^ = 54.30	< 0.001
< $ 20,000	23,930 (85.87)	11,537 (87.48)	12,393 (84.22)		
≥ $ 20,000	6,504 (14.13)	2,593 (12.52)	3,911 (15.78)		
Alcohol drinking, n (%)	20,969 (74.00)	10,122 (76.04)	10,847 (71.91)	χ^2^ = 27.60	< 0.001
Smoking history, n (%)	13,904 (45.56)	5,849 (41.66)	8,055 (49.56)	χ^2^ = 93.84	< 0.001
Diabetes, n (%)	4,033 (9.83)	722 (3.63)	3,311 (16.19)	χ^2^ = 812.84	< 0.001
Stroke, n (%)	1,137 (2.78)	162 (0.91)	975 (4.71)	χ^2^ = 262.64	< 0.001
Trouble sleeping, n (%)	7,845 (27.74)	2,842 (22.18)	5,003 (33.45)	χ^2^ = 191.64	< 0.001
Depression, n (%)	2,641 (7.54)	1,063 (6.45)	1,578 (8.65)	χ^2^ = 31.91	< 0.001
Sleep duration, hours, Mean (S.E.)	7.11 (0.01)	7.12 (0.02)	7.09 (0.02)	t = 1.74	0.085

*BMI* body mass index, *GED* general educational development

### Distribution of people with and without hypertension

Age (55 years old vs. 40 years old, *P* < 0.001) and BMI (30.77 kg/m^2^ vs. 27.41 kg/m^2^, *P* < 0.001) of individuals with hypertension were higher than those of individuals without hypertension, as shown in Table 1. Moreover, the proportions of smoking history (49.56% vs. 41.66%, *P* < 0.001), diabetes (16.19% vs. 3.63%, *P* < 0.001), stroke (4.71%, vs. 0.91% *P* < 0.001), trouble sleeping (33.45% vs. 22.18%, *P* < 0.001) and depression (8.65% vs. 6.45%, *P* < 0.001) in participants with hypertension were higher than those in participants without hypertension. In addition, the distribution of gender, race, marital status, education level, annual family income and alcohol drinking in the hypertension group were different from those in the non-hypertension group, and the difference was statistically significant (*P* < 0.001).

### Association between trouble sleeping and hypertension

**Fig. 2:**
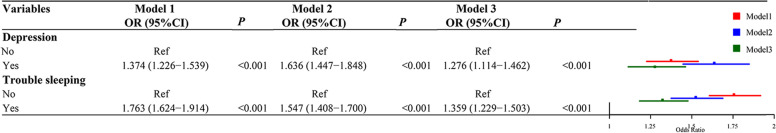
Multivariate logistic regression of trouble sleeping and depression for people with hypertension (Ref, reference; OR, odds ratio; CI, confidence interval; Model 1, unadjusted model; Model 2, adjustment for age and gender; Model 3, adjustment for age, gender, BMI, race, marital status, education, annual family income, alcohol drinking, smoking history, diabetes, stroke and sleep duration)

Compared to those who did not have trouble sleeping, those who had trouble sleeping were positively associated with the risk of hypertension [crude odd ratio (OR) with 95% confidence interval (CI) of 1.763 (1.624–1.914) and OR of 1.547 (95% CI: 1.408–1.700) in Model 2]. After adjustment for age, gender, BMI, race, marital status, education, annual family income, alcohol drinking, smoking history, diabetes stroke, and sleep duration, those who had trouble sleeping had a higher risk of hypertension [OR = 1.359 (95% CI: 1.229–1.503)] than those without trouble sleeping. The positive association between trouble sleeping and hypertension is shown in Fig. 2.

### Association between depression and hypertension

Participants with depression were positively associated with the risk of hypertension compared to those without depression [adjusted OR of 1.276 (95% CI: 1.114–1.462)] in Model 3. The positive association between depression and hypertension is shown in Fig. 2.

### Interaction between trouble sleeping and depression on hypertension

**Fig. 3:**
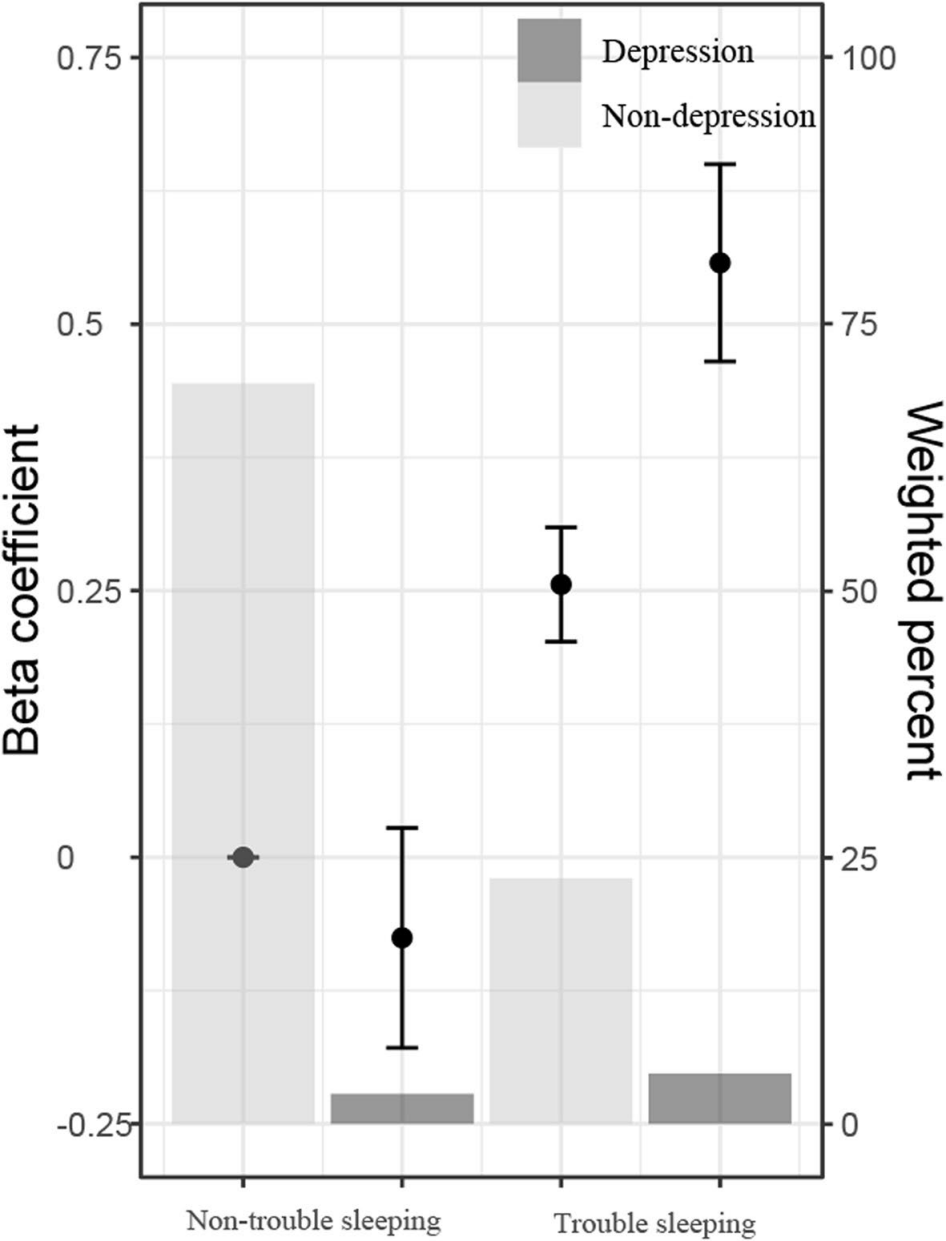
Interaction between trouble sleeping and depression in Model 3 (Model 3: adjustment for age, gender, BMI, race, marital status, education, annual family income, alcohol drinking, smoking history, diabetes, stroke and sleep duration; bar graph: weighted percentage within each joint subgroup; dot plot: beta coefficient; error bars: 95% confidence intervals)

Results in Table 2 indicated that there was a significant synergistic effect of trouble sleeping and depression on hypertension in Model 3 (adjusted RERI = 0.528, 95% CI = 0.182–0.873; adjusted AP = 0.302, 95% CI = 0.140–0.465; adjusted S = 3.413, 95% CI = 1.301–8.951). Among them, the value of AP was 0.302 in Model 3, indicating that 30.2% of hypertension cases were caused by the interaction between trouble sleeping and depression in the samples of this study. Consistently, Fig. 3 showed there was an additive interaction between trouble sleeping and depression on hypertension, which was similar to the presented interaction in a published article [29].

**Table 2:** Interactive effect analysis of trouble sleeping and depression

Trouble sleeping	Depression	Hypertension/Total (n)	Model 1			Model 2			Model 3		
			OR	95% CI	*P*	OR	95% CI	*P*	OR	95% CI	*P*
0	0	10,751/21,488	Ref			Ref			Ref		
0	1	551/1,101	0.962	0.814–1.137	0.548	1.183	0.985–1.421	0.071	0.927	0.756–1.137	0.465
1	0	3,975/6,305	1.696	1.558–1.847	< 0.001	1.437	1.300–1.589	< 0.001	1.291	1.161–1.436	< 0.001
1	1	1,028/1,540	2.120	1.800–2.496	< 0.001	2.298	1.937–2.726	< 0.001	1.746	1.454–2.098	< 0.001
RERI (95%CI)			0.461 (0.099–0.823)			0.678 (0.269–1.086)			0.528 (0.182–0.873)		
AP (95%CI)			0.217 (0.074–0.361)			0.295 (0.155–0.435)			0.302 (0.140–0.465)		
S (95%CI)			1.700 (1.142–2.530)			2.093 (1.359–3.222)			3.413 (1.301–8.951)		

*Ref* reference, *RERI* relative excess risk due to interaction, *AP* attributable proportion of interaction, *S* synergy index, *OR* odd ratio, *CI* confidence interval

Model1: unadjusted model

Model2: adjustment for age and gender

Model3: adjustment for age, gender, BMI, race, marital status, education, annual family income, alcohol drinking, smoking history, diabetes, stroke and sleep duration

### Interaction between trouble sleeping and depression severity on hypertension

The moderate depression was the only variable that remained significant in Model 3 with RERI = 0.614 (95% CI: 0.214–1.014), AP = 0.347 (95% CI: 0.172–0.523), and S = 4.969 (95% CI: 1.022–24.155), suggesting that there was a synergistic interaction between moderate depression and trouble sleeping in people with hypertension (Table 3).

**Table 3:** Interactive effect analysis of trouble sleeping and depression severity

Trouble sleeping	Depression	Moderate depression			Moderately severe depression			Severe depression		
		OR	95% CI	*P*	OR	95% CI	*P*	OR	95% CI	*P*
0	0	Ref			Ref			Ref		
0	1	0.868	0.694–1.087	0.216	0.987	0.655–1.488	0.951	1.219	0.552–2.695	0.621
1	0	1.286	1.157–1.430	< 0.001	1.287	1.157–1.433	< 0.001	1.290	1.160–1.435	< 0.001
1	1	1.769	1.444–2.167	< 0.001	1.749	1.237–2.473	< 0.001	1.576	1.046–2.372	< 0.001
RERI (95%CI)		0.614 (0.214–1.014)			0.474 (-0.216 – 1.164)			0.066 (-1.016—1.148)		
AP (95%CI)		0.347 (0.172–0.523)			0.271 (-0.056 – 0.598)			0.042 (-0.638—0.722)		
S (95%CI)		4.969 (1.022–24.155)			2.725 (0.520 – 14.291)			1.129 (0.143 – 8.914)		

Reference (Ref.). *Ref* reference, *RERI* relative excess risk due to interaction, *AP* attributable proportion of interaction, *S* synergy index, *OR* odd ratio, *CI* confidence interval

## Discussion

In the present study, we utilized NHANES 2005–2018 data of the included 30,434 people to study the associations of trouble sleeping and depression with hypertension and their interactions on hypertension. We found that there was a synergistic interaction between trouble sleeping and depression, especially moderate depression, on hypertension.

Our present study observed a significant positive association between trouble sleeping and hypertension in the whole study population after adjustment for age, gender, BMI, race, marital status, education, annual family income, alcohol drinking, smoking history, diabetes, stroke and sleep duration. The association between trouble sleeping and hypertension has been proposed and explored previously. Trouble sleeping includes obstructive sleep apnea (OSA), sleep quality (sleep deprivation, sleep duration and insomnia), and combinations of sleep problems. Studies demonstrated the high incidence of hypertension in patients with OSA [30], and the blood pressure seemed to be dose-related to OSA [31]. OSA-related hypertension is usually attributed to increased diastolic blood pressure caused by sympathetic activation, which stimulated the renin angiotensin-aldosterone system to increase vascular resistance and cardiac output, causing hypertension [32, 33].

Short sleep duration may be representative of sleep disorder, and was significantly associated with hypertension [34, 35], so we adjusted sleep duration in the multivariate logistic regression model. Previous studies showed that short sleep duration (less than 6 h) had an increased risk of hypertension compared to 7 h in a cross-study analysis of more than 7000 samples [34], and a meta-analysis by Osamu et al. had similar results [35]. This may be due to short sleep breaks disrupting circadian rhythm and autonomic balance [36]. Another hypothesis suggested that sleep deprivation or short sleep duration was the stress state that has been shown to promote salt appetite, and inhibit the excretion of renal salt fluid [37], and excessive salt intake has been confirmed to be a risk factor for hypertension. Notably, long sleep duration (≥ 10 h) was also considered as a risk factor of hypertension [34]. Guo et al. also found that long sleep duration was positively associated with hypertension in a systematic review and meta-analysis [38]. Most studies involving sleep problems have used self-reported data. Compared with healthy people, people with functional limitations due to chronic diseases may spend more time in bed, and it may be mistakenly reported as sleep and cause confusion about health condition. Herein, although sleep duration seemed to be related to the prevalence and incidence of hypertension, most cross-sectional studies to date have failed to demonstrate any causality. Moreover, a large population-based study observed significant associations between hypertension and poor sleep quality based on the Pittsburgh Sleep Quality Index (PSQI) scores in rural China [39]. Rahim et al. found an association between history of shift work (poor sleep quality) and rates of hypertension in 7,420 Ontario workers aged 35 to 69 years in a 12-year longitudinal cohort study [40]. Thus, changes in the sleep quality or quantity could lead to loss of blood pressure dipping pattern and increased sympathetic activity at night, and then a continuous increase in sympathetic tone in turn, which could result in hypertension [41].

Trouble sleeping could exist as a single disease, but generally coexist with physical or mental diseases, and long-term sleep quality decline could cause hypertension, low immunity and psychological disorders [42]. Xiuli Song et al. indicated that there was a significant association between depression and elevated blood pressure using support vector machine (SVM) [43]. Two cohort studies found that depression was the risk factor of hypertension [44, 45], which was consistent with our results that depression was significantly positively associated with hypertension in the fully adjusted model. Prior studies found that people with hypertension often have sleep problems and depression [46, 47]. In addition, it was uncovered that depression was a trigger for hypertension, and hypertension could easily make depression worse [48]. Patients with hypertension were prone to anxiety, depression and other adverse emotions due to repeated illness, and these adverse emotions induced the fluctuation of blood pressure in patients, causing a vicious circle [47, 49]. Therefore, it was of great significance to take reasonable and effective nursing methods to keep the mood cheerful and blood pressure stable in patients with hypertension.

Many studies reported that anxiety, depression, tension and depressive emotions were not only predisposing factors for hypertension, but also important factors affecting the sleep quality of hypertensive patients [50, 51]. A previous study showed that insomnia was associated with mental and psychosomatic disorders [41], and pure insomnia and insomnia comorbid with depression had a strong correlation in a longitudinal cohort study [52]. Additionally, trouble sleeping was a risk factor for people with hypertension. A longitudinal population-based study from 2008 to 2016 identified that insomnia may contribute to the comorbidity of hypertension and depression in the United States [53]. Herein, we found similar results that there was a synergistic interaction between trouble sleeping and depression on hypertension. In normal circumstances, happiness, emotional stability and life satisfaction were regarded as positive factors for maintaining sleep quality, while lack of happiness, tension, depression and anger in life were regarded as negative factors affecting sleep quality [50, 51]. The bad mood could easily lead to trouble sleeping, which seriously endangered physical and mental health. Moreover, when subgroup analysis was conducted, we only found this synergistic effect between moderate depression and trouble sleeping on hypertension. This may be because the synergistic effect of moderate depression and trouble sleeping was more significant than that of moderately severe or severe depression and trouble sleeping. Although there was no statistical significance between them, the estimated joint effect of two factors was greater than the independent effect of trouble sleeping and moderately severe/severe depression, respectively. Therefore, it was suggested that clinicians should actively intervene in psychological disorders, especially depressive symptoms, while paying attention to improving the sleep quality of patients in the routine drug treatment of hypertension.

Our research strengths lied in the following aspects. First, we used seven cycles, high-quality, representative data from the NHANES about Americans, which was large and multistage. Second, previous studies showed that trouble sleeping and depression were independent risk factors for hypertension, but few studies indicated an interaction between them on hypertension. Here, we found that the synergistic interaction between trouble sleeping and depression on hypertension. Third, the logistic regression model was adjusted for several potential confounding factors, including demographic, socioeconomic, health and lifestyle information, etc.

Several potential limitations existed in this study. Firstly, the measurement of trouble sleeping in this study was self-reported, it was subjective rather than objective. It was also likely that there was no comprehensive understanding of the participants’ sleep quality, sleep efficiency, sleep duration, etc. Perhaps objective measurement methods for sleep health such as activity scanners and polysomnography were needed to strengthen investigations in this field. Secondly, it was a cross-sectional survey, with compromised accuracy and generalizability, so we could not determine the causal relationship or exclude bidirectional relationships.

## Conclusion

Our results indicated that trouble sleeping and depression had a synergistic interaction on hypertension. In depression subgroup analysis, moderate depression and trouble sleeping had a significant synergistic effect on hypertension. This suggested that efforts should also be made to improve patients' psychological status and trouble sleeping in addition to actively and effectively controlling the primary disease. Our results may provide epidemiological evidence for the independent association of trouble sleeping and depression with hypertension and the interaction between trouble sleeping and depression on hypertension.

## Data Availability

More information about the NHANES could be obtained at: http://www.cdc.gov/nhanes.

## Abbreviations

NHANES: National Health and Nutritional Examination Survey
RERI: Relative excess risk due to interaction
AP: Attributable proportion of interaction
S: Synergy index
NCHS: National Center for Health Statistics
CDC: Centers for Disease Control and Prevention
CAPI: Computer-Assisted Personal Interview
PHQ-9: Patient Health Questionnaire

## References

[1] Whelton P, Carey R, Aronow W, Casey D, Collins K, Dennison Himmelfarb C, DePalma S, Gidding S, Jamerson K, Jones D, et al. 2017 ACC/AHA/AAPA/ABC/ACPM/AGS/APhA/ASH/ASPC/NMA/PCNA Guideline for the prevention, detection, evaluation, and management of high blood pressure in adults: executive summary: a report of the American college of cardiology/American heart association task force on clinical practice guidelines. Hypertension. 2018;71(6):1269–324 (Dallas, Tex : 1979). 10.1161/HYP.0000000000000066 29133354

[2] Rossier BC, Bochud M, Devuyst O (2017). The Hypertension Pandemic: An Evolutionary Perspective. Physiology (Bethesda).

[3] Fisher N, Curfman G. Hypertension—A Public Health Challenge of Global Proportions. JAMA. 2018;320(17):1757–59. 10.1001/jama.2018.16760 30398584

[4] Rahimi K, Emdin CA, Macmahon S (2015). The Epidemiology of Blood Pressure and Its Worldwide Management. Circ Res.

[5] Angeli F, Reboldi G, Trapasso M, Gentile G, Pinzagli MG, Aita A (2019). European and US guidelines for arterial hypertension: similarities and differences. Eur J Intern Med.

[6] Chen J, Zhang C, Wu Y, Zhang D. Association between Hypertension and the Risk of Parkinson's Disease: A Meta-Analysis of Analytical Studies. Neuroepidemiology. 2019;52:181–92. 10.1159/000496977 30726850

[7] Han H, Guo W, Shi W, Yu Y, Zhang Y, Ye X (2017). Hypertension and breast cancer risk: a systematic review and meta-analysis. Sci Rep.

[8] Ford ES, Cunningham TJ, et al. Trends in Self-Reported Sleep Duration among US Adults from 1985 to 2012. Sleep. 2015;38:829–32. 10.5665/sleep.4684 PMC4402659 25669182

[9] St-Onge MP, Grandner MA, Brown D, Conroy MB, Jean-Louis G, Coons M, et al. Sleep Duration and Quality: Impact on Lifestyle Behaviors and Cardiometabolic Health: A Scientific Statement From the American Heart Association. Circulation. 2016;134:e367–86. 10.1161/CIR.0000000000000444 PMC5567876 27647451

[10] Yin J, Jin X, Shan Z, et al. Relationship of Sleep Duration With All‐Cause Mortality and Cardiovascular Events: A Systematic Review and Dose‐Response Meta‐Analysis of Prospective Cohort Studies. J Am Heart Assoc. 2017;6(9):e005947. 10.1161/JAHA.117.005947 PMC5634263 28889101

[11] Javaheri S, Redline S (2017). Insomnia and Risk of Cardiovascular Disease. Chest.

[12] Thomas SJ, Calhoun D (2017). Sleep, insomnia, and hypertension: current findings and future directions. J Am Soc Hypertens.

[13] Palagini L, Bruno RM, Gemignani A, Baglioni C, Ghiadoni L, Riemann D. Sleep Loss and Hypertension: A Systematic Review. Curr Pharm Des. 2013;19(13):2409–19. 10.2174/1381612811319130009 23173590

[14] Jihye K, Inho J (2010). Age-Dependent Association Between Sleep Duration and Hypertension in the Adult Korean Population. Am J Hypertens.

[15] Ramos AR, Jin Z, Rundek T, Russo C, Homma S, Elkind M (2013). Relation between long sleep and left ventricular mass (from a multiethnic elderly cohort). Am J Cardiol.

[16] Hammen C (2018). Risk Factors for Depression: An Autobiographical Review. Annu Rev Clin Psychol.

[17] Bergantin LB (2020). Depression Rises the Risk of Hypertension Incidence: Discussing the Link through the Ca2+/cAMP Signalling. Curr Hypertens Rev.

[18] Lett HS, Blumenthal JA, Babyak MA, Sherwood A, Strauman T, Robins C (2004). Depression as a risk factor for coronary artery disease: evidence, mechanisms, and treatment. Psychosom Med.

[19] Axon RN, Zhao Y, Egede LE (2010). Association of depressive symptoms with all-cause and ischemic heart disease mortality in adults with self-reported hypertension. Am J Hypertens.

[20] Morin CM, Jarrin DC. Epidemiology of Insomnia : Prevalence, Course, Risk Factors, and Public Health Burden. Sleep Med Clin. 2013;8(3):281–97. 10.1016/j.jsmc.2022.03.003 35659072

[21] Centers for Disease Control and Prevention. National Health and Nutrition Examination Survey, 2007-2008: overview, HHS, Centers for Disease Control and Prevention, National Center for Health Statistics, Hyattsville, MD (2007) [Cited 2021 June 29]. Available from: https://www-cdc-gov.libproxy.library.unt.edu/nchs/data/nhanes/nhanes_07_08/overviewbrochure_0708.pdf.

[22] Cao C, Cade W, Li S, McMillan J, Friedenreich C, Yang L (2021). Association of Balance Function With All-Cause and Cause-Specific Mortality Among US Adults. JAMA..

[23] Cao C, Yang L, Cade W, Racette S, Park Y, Cao Y, Friedenreich C, Hamer M, Stamatakis E, Smith L (2020). Cardiorespiratory Fitness Is Associated With Early Death Among Healthy Young and Middle-Aged Baby Boomers and Generation Xers. Am J Med.

[24] Cao C, Hu L, Xu T, Liu Q, Koyanagi A, Yang L, Carvalho A, Cavazos-Rehg P, Smith L (2020). Prevalence, correlates and misperception of depression symptoms in the United States, NHANES 2015–2018. J Affect Disord.

[25] Giles WH, Croft JB, Keenan NL, Lane MJ, Wheeler FC (1995). The Validity of Self-reported Hypertension and Correlates of Hypertension Awareness Among Blacks and Whites Within the Stroke Belt. Am J Prev Med.

[26] Martin LM, Leff M, Calonge N, Garrett C, Nelson DE (2000). Validation of self-reported chronic conditions and health services in a managed care population. Am J Prev Med.

[27] Kroenke K, Spitzer RL, Williams J (2001). The PHQ-9: validity of a brief depression severity measure. J Gen Intern Med.

[28] Jorgensen D, White GE, Sekikawa A, Gianaros P. Higher dietary inflammation is associated with increased odds of depression, independent of Framingham Risk Score in the National Health and Nutrition Examination Survey. Nutr Res. 2018;54:23–32. 10.1016/j.nutres.2018.03.004 PMC6011232 29914664

[29] Liao J, Cao C, Hur J, Cohen J, Chen W, Zong X, Colditz G, Yang L, Stamatakis E, Cao Y (2021). Association of sedentary patterns with body fat distribution among US children and adolescents: a population-based study. Int J Obes.

[30] Hou H, Zhao Y, Yu W, Dong H, Xue X, Ding J (2018). Association of obstructive sleep apnea with hypertension: A systematic review and meta-analysis. J Glob Health..

[31] Lavie P (2000). Obstructive sleep apnoea syndrome as a risk factor for hypertension: population study. BMJ.

[32] Calhoun DA, Harding SM (2010). Sleep and hypertension. Chest.

[33] Baguet JP, Hammer L, Lévy P, Pierre H, Rossini E, Mouret S (2005). Night-time and diastolic hypertension are common and underestimated conditions in newly diagnosed apnoeic patients. J Hypertens.

[34] Grandner M, Mullington JM, Hashmi SD, Redeker NS, Watson NF, Morgenthaler TI (2018). Sleep Duration and Hypertension: Analysis of > 700,000 Adults by Age and Sex. J Clin Sleep Med.

[35] Itani O, Jike M, Watanabe N, Kaneita Y (2017). Short sleep duration and health outcomes: a systematic review, meta-analysis, and meta-regression. Sleep Med.

[36] Kreier F, Yilmaz A, Kalsbeek A, Romijn JA, Sauerwein HP, Fliers E (2003). Hypothesis: Shifting the Equilibrium From Activity to Food Leads to Autonomic Unbalance and the Metabolic Syndrome. Diabetes.

[37] Folkow B. Mental Stress and its Importance for Cardiovascular Disorders; Physiological Aspects, “From-Mice-to-Man.” Scand Cardiovasc J. 2001;35(3):163–72. 11515688

[38] Guo X, Zheng L, Wang J, Zhang X, Zhang X, Li J (2013). Epidemiological evidence for the link between sleep duration and high blood pressure: a systematic review and meta-analysis. Sleep Med.

[39] Liu RQ, Qian Z, Trevathan E, Chang JJ, Zelicoff A, Hao YT (2016). Poor sleep quality associated with high risk of hypertension and elevated blood pressure in China: results from a large population-based study. Hypertens Res.

[40] Rahim A, McIsaac MA, Aronson KJ, Smith PM, Tranmer JE (2021). The Associations of Shift Work, Sleep Quality, and Incidence of Hypertension in Ontario Adults: A Population-Based Study. Can J Cardiol.

[41] Pepin JL, Borel AL, Tamisier R, Baguet JP, Levy P, Dauvilliers Y (2014). Hypertension and sleep: overview of a tight relationship. Sleep Med Rev.

[42] Tobaldini E, Costantino G, Solbiati M, Cogliati C, Kara T, Nobili L (2017). Sleep, sleep deprivation, autonomic nervous system and cardiovascular diseases. Neurosci Biobehav Rev.

[43] Song X, Zhang Z, Zhang R, Wang M, Lin D, Li T (2018). Predictive markers of depression in hypertension. Medicine (Baltimore)..

[44] Sandstrom YK, Ljunggren G, Wandell P, Wahlstrom L, Carlsson AC (2016). Psychiatric comorbidities in patients with hypertension–a study of registered diagnoses 2009–2013 in the total population in Stockholm County. Sweden. J Hypertens..

[45] Stenman M, Holzmann MJ, Sartipy U (2014). Relation of major depression to survival after coronary artery bypass grafting. Am J Cardiol.

[46] Kielbasa G, Stolarz-Skrzypek K, Pawlik A, Latka M, Drozdz T, Olszewska M (2016). Assessment of sleep disorders among patients with hypertension and coexisting metabolic syndrome. Adv Med Sci.

[47] Chen S, Conwell Y, Xue J, Li LW, Tang W, Bogner HR (2018). Protocol of an ongoing randomized controlled trial of care management for comorbid depression and hypertension: the Chinese Older Adult Collaborations in Health (COACH) study. BMC Geriatr.

[48] Kretchy IA, Owusu-Daaku F, Danquah SA (2014). Mental health in hypertension: assessing symptoms of anxiety, depression and stress on anti-hypertensive medication adherence. Int J Ment Health Syst.

[49] Li Z, Li Y, Chen L, Chen P, Hu Y (2015). Prevalence of Depression in Patients With Hypertension: A Systematic Review and Meta-Analysis. Medicine (Baltimore)..

[50] Ma L, Li Y (2017). The effect of depression on sleep quality and the circadian rhythm of ambulatory blood pressure in older patients with hypertension. J Clin Neurosci.

[51] Bussotti M, Sommaruga M (2018). Anxiety and depression in patients with pulmonary hypertension: impact and management challenges. Vasc Health Risk Manag.

[52] Buysse DJ, Angst J, Gamma A, Ajdacic V, Eich D, Rössler W (2008). Prevalence, Course, and Comorbidity of Insomnia and Depression in Young Adults. Sleep.

[53] Dong Y, Yang FM (2019). Insomnia symptoms predict both future hypertension and depression. Prev Med.

